# Graphene Oxide/Ferrocene-Containing Polymer/Gold Nanoparticle Triple Nanocomposite

**DOI:** 10.3390/nano9020310

**Published:** 2019-02-25

**Authors:** Wenhao Qian, Tao Song, Mao Ye, Haiyan Zhang, Chun Feng, Guolin Lu, Xiaoyu Huang

**Affiliations:** 1 Department of Stomatology, Shanghai Xuhui District Dental Center, 685 Zhaojiabang Road, Shanghai 200032, China; songtao21@139.com (T.S.); doreye@139.com (M.Y.); 13641956377@163.com (H.Z.); 2 Key Laboratory of Synthetic and Self-Assembly Chemistry for Organic Functional Molecules, Center for Excellence in Molecular Synthesis, Shanghai Institute of Organic Chemistry, Chinese Academy of Sciences, 345 Lingling Road, Shanghai 200032, China; cfeng@sioc.ac.cn (C.F.); luguolin@sioc.ac.cn (G.L.)

**Keywords:** graphene oxide, ferrocene, ATRP, disulfide bond, gold nanoparticle

## Abstract

A facile strategy to prepare GO-based nanocomposites with both gold nanoparticles (AuNPs) and ferrocene (Fc) moieties was developed. The surface of GO was modified with PFcMAss homopolymer by surface-initiated atom transfer radical polymerization of a new methacrylate monomer of 2-((2-(methacryloyloxy)ethyl)disulfanyl)ethyl ferrocene-carboxylate (FcMAss), consisting of disulfide as an anchoring group for stabilizing AuNPs and Fc group as an additional functionality. AuNPs with an average diameter of about 4.1 nm were formed *in situ* on the surface of PFcMAss-decorated GO (GO-PFcMAss) via Brust-Schiffrin method to give GO-PFcMAss-AuNPs multifunctional nanocomposites bearing GO, AuNPs and Fc groups. The obtained nanocomposites were characterized by X-ray photoelectron spectroscopy (XPS), X-ray diffraction (XRD), transmission electron microscopy (TEM) and atomic force microscopy (AFM). Since disulfide-containing polymers, rather than the commonly used thiol-containing compounds, were employed as ligands to stabilize AuNPs, much more stabilizing groups were attached onto the surface of GO, and thus more AuNPs were able to be introduced onto the surface of GO. Besides, polymeric chains on the surface of GO endowed GO-PFcMAss-AuNPs nanocomposites with excellent colloidal stability, and the usage of a disulfide group provides possibility to efficiently incorporate additional functionalities by easily modifying structure of disulfide-based monomer.

## 1. Introduction

Graphene, a fascinating two-dimensional graphitic carbon system, has quickly sparked tremendous interests across many fields including biosensors, electrochemical energy storage and electronics due to its extraordinary properties of large surface area, good mechanical properties and extremely high electronic conductivity properties [1,2,3,4,5,6]. Graphene oxide (GO), a main derivative of graphene, offers great potential for large-scale production of graphene-based materials by taking advantage of carboxyl, hydroxyl and epoxy groups on the surface of GO. As GO can be prepared efficiently in large scale and low-cost fashion from cheap graphite by chemical methods [7,8,9,10], it is considered as a star building motif for the construction of graphene-based composite materials. Up to now, a large variety of functional moieties, including inorganic particles, polymers, metal-organic frameworks (MOFs), carbon nanotubes (CNTs) and so on, have been incorporated onto the surface of GO to improve inherent properties of GO or endow GO-based hybrid materials with new functionalities [11,12,13,14,15,16,17,18,19,20,21,22,23]. Although significant advances have been achieved in the preparation of GO-based hybrid materials, increasing attention has been still paid on exploring more facile and robust strategies to efficiently introduce diverse functionalities onto the surface of GO for the fabrication of hierarchically multi-functional GO-based composite materials.

Gold nanoparticles (AuNPs), one of the most attractive inorganic nanoparticles, have attracted intense interest for applications in photonics, information storage, electronic and optical detection system, therapeutics, diagnostics, photovoltatics and catalysis because of their unique properties, such as high surface free energy, strong adsorption ability, distinctive optical and electrical properties, and excellent biocompatibility [24,25,26,27,28,29,30,31]. Therefore, various composites consisting of AuNPs and GO have been fabricated to prepare desired multi-functional materials, in which functionalities of AuNPs and GO were combined together. The synergetic effect would not only lead to the improvement of performance of composites, but also endow the composites with some new functionalities, which cannot be gained by either GO or AuNPs separately [32,33,34,35,36,37,38]. In previous reports, AuNPs were anchored onto the surface of GO via either noncovalent interactions including van der Waals interaction, hydrogen bonding, π-π stacking, electrostatic interactions, or covalent bonds [32,33,34,35,36,37,38]. Since the noncovalent interactions can be easily weakened by temperature, salt, pH or some specific compounds, the limited stability of GO/AuNPs composites would hinder the application of these composites. Although the stability can be significantly improved by using covalent bonds instead of noncovalent interactions, only a few approaches were developed [39,40,41,42,43]. For these approaches, the linkers between AuNPs and GO were small molecules with a thiol group, where theoretically one reactive site of GO only can be used to install one thiol group. But a large number of thiol-based ligands are generally necessary to stabilize one gold nanoparticle. Thus, the accessibility of GO surface to AuNPs was highly retarded. In addition, these methods were not efficient for the incorporation of additional functional moieties.

**Scheme 1:**
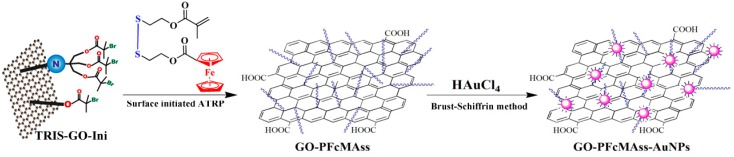
Preparation of GO-PFcMAss-AuNPs nanocomposite.

Ferrocene (Fc) is an interesting metal-containing compound with unique redox, preceramic, etch-resistant and catalytic properties [44,45,46]. Thus, Fc and its derivatives are considered to be attractive building blocks for the preparation of functional materials. For example, Fc has been widely used to construct electrochemical biosensors due to its reversibility, regeneration at low potential and generation of stable redox states [44,45,46]. Ferrocene and its derivatives, as excellent redox mediators, have been well studied in electrochemical sensing. However, ferrocene cannot form stable adsorption layers on electrodes so that enormous efforts have been performed to immobilize ferrocene and its derivatives on electrode. As it is well known, various nanomaterials, such as AuNPs, carbon nanotubes and graphene, have been used as excellent carriers to enhance the sensitivity of sensors. Herein, we reported a facile strategy to prepare GO-based nanocomposites with both AuNPs and Fc moieties as shown in Scheme 1. We firstly designed and synthesized a new methacrylate monomer of 2-((2-(methacryloyloxy)ethyl)disulfanyl)ethyl ferrocenecarboxylate (FcMAss) with disulfide and Fc groups, in which disulfide group can serve as the anchoring group for AuNPs and Fc group can act as an additional functional unit. Subsequently, poly(2-((2-(methacryloyloxy)ethyl)disulfanyl)ethyl ferrocenecarboxylate) (PFcMAss) was introduced onto the surface of GO by surface-initiated atom transfer radical polymerization (SI-ATRP) [47,48]. Then, by the virtue of the strong coordinating ability of disulfide moieties toward gold nanoparticles, gold nanoparticles with an average diameter of 4.1 nm were formed *in situ* on the surface of PFcMAss-decorated GO (GO-PFcMAss) via Brust-Schiffrin method [49] to give a multi-functional nanocomposite of GO-PFcMAss-AuNPs consisting of GO, AuNPs and Fc groups. In the current case, disulfide-containing polymers covalently connected to GO can be employed as ligands to stabilize AuNPs and simultaneously introduce Fc groups onto the surface of AuNPs. Therefore, more AuNPs and Fc groups are able to be immobilized onto the surface of GO, which allows the amplification of detection signal. Thus, the obtained nanomaterials combining ferrocene, gold nanoparticles and GO together, may allow the development of a signal amplification detection platform for electro-active molecules. The obtained GO-PFcMAss-AuNPs nanocomposite was characterized by X-ray photoelectron spectroscopy (XPS), X-ray diffraction (XRD), transmission electron microscopy (TEM) and atomic force microscopy (AFM).

## 2. Experimental

### 2.1. Characterization

FT-IR spectra were recorded on a Nicolet AVATAR-360 FT-IR spectrophotometer (Waltham, MA, USA) with a resolution of 4 cm^−1^. ^1^H-NMR measurements were performed on a JEOL resonance ECZ 400S s (400 MHz) pectrometer (Tokyo, Japan) in CDCl_3_ using tetramethylsilane (TMS) as an internal standard. Electrospray ionization mass spectrometry (ESI-MS) was measured by an Agilent FTMS-7.0 Fourier transformation mass spectrometer (Santa Clara, CA, USA). Relative molecular weight and molecular weight distribution of PFcMAss homopolymer were measured by a conventional gel permeation chromatography (GPC) system equipped with a Waters 1515 Isocratic HPLC pump (Milford, MA, USA), a Waters 2414 refractive index detector (Milford, MA, USA) and a set of Waters Styragel columns (HR3 (500–30,000), HR4 (5000–600,000) and HR5 (50,000–4,000,000), 7.8 × 300 mm, particle size: 5 μm). GPC measurement was carried out at 35 °C using THF as eluent with a flow rate of 1.0 mL/min. The system was calibrated with linear poly(methyl methacrylate) standards. Powder X-ray diffraction (XRD) measurements were run by a Philips X’Pert PRO X-ray powder diffractometer (Amsterdam, Netherlands) with Cu*Kα* (1.541 Å) radiation (40 kV, 40 mA), the samples were exposed at a scan rate of 2θ = 0.04244°/s in the range of 5° and 60°. X-ray photoelectron spectroscopy (XPS) was recorded on a PHI 5000c ESCA photoelectron spectrometer (Waltham, IN, USA). Transmission electron microscopy (TEM) images were obtained by a JEOL JEM 1230 instrument (Tokyo, Japan) operated at 80 kV. Atomic force microscopy (AFM) images were taken by a Veeco DI MultiMode SPM (Plainview, TX, USA) in the tapping mode of dropping the sample solution onto the freshly exfoliated mica substrate.

### 2.2. Preparation of 2-((2-(Methacryloyloxy)ethyl)disulfanyl)ethyl Ferrocene-carboxylate (FcMAss)

**Scheme 2:**
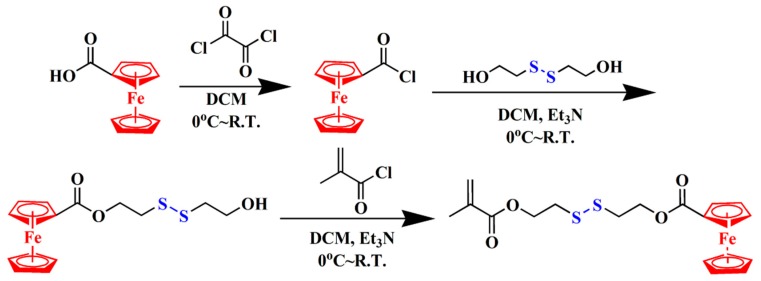
Synthesis of FcMAss monomer.

Dichloromethane (DCM) was dried over CaH_2_ and distilled over CaH_2_ under N_2_ prior to use. Triethylamine (Et_3_N, Aldrich, 99.5%) was dried over KOH and distilled over CaH_2_ under N_2_ prior to use. As shown in Scheme 2, ferrocenoyl chloride obtained by reacting ferrocenecarboxylic acid (Alfa Aesar (Shanghai, China), 98%) with oxalyl chloride (J&K, Beijing, China, 98%) was treated with bis(2-hydroxyethyl) disulfide (Alfa Aesar, 90%) to afford the monoesterification product, which reacted with methacryloyl chloride (Aldrich, St. Louis, MO, USA, 90%) in the presence of Et_3_N to produce the target monomer, FcMAss (total yield: 53.4%). FI-IR (film): *ν* (cm^−1^): 2960, 2858, 1716, 1637, 1460, 1380, 1273, 1218, 1132, 1068, 941, 912, 818, 773. ^1^H-NMR (400 MHz, CDCl_3_): *δ* (ppm): 1.95 (3H, C*H*_3_C), 3.02 (4H, CO_2_CH_2_C*H*_2_S–SC*H*_2_CH_2_O_2_C), 4.21, 4.40, 4.81 (9H, ferrocenyl), 4.45 (4H, CO_2_C*H*_2_CH_2_S-SCH_2_C*H*_2_O_2_C), 5.58, 6.14 (2H, C*H*_2_=C).

### 2.3. Preparation of PFcMAss via ATRP of FcMAss

**Scheme 3:**
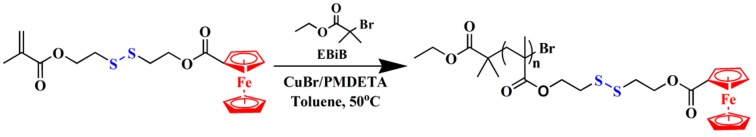
ATRP of FcMAss monomer.

Toluene (Aldrich, 99%) were dried over CaH_2_ and distilled from sodium and benzophenone under N_2_ prior to use. Copper(I) bromide (CuBr, Aldrich, 98%) was purified by stirring over CH_3_COOH overnight at room temperature, followed by washing with ethanol, diethyl ether and acetone prior to drying at 40 °C *in vacuo* for one day. As shown in Scheme 3, CuBr (5.8 mg, 0.04 mmol) and FcMAss (520.8 mg, 1.2 mmol) were first added to a 25 mL Schlenk flask (flame-dried under vacuum prior to use) sealed with a rubber septum. After evacuating and backfilling with Ar, pentamethyldiethylenetriamine (PMDETA, Aldrich, 99%, 8.5 μL, 0.04 mmol), ethyl α-bromoisobutyrate (EBiB, Aldrich, 98%, 6 μL, 0.04 mmol) and dry toluene (3 mL) were introduced via a gastight syringe followed by three cycles of freezing-pumping-thawing. The flask was immersed into an oil bath set at 50 °C. After 48 h, the polymerization was terminated by immersing the flask into liquid N_2_. The reaction mixture was diluted with THF and filtered through a short Al_2_O_3_ column to remove the residual copper complex. THF was rotary evaporated and the crude product was purified by dissolving in THF and precipitating in cold *n*-hexane/diethyl ether (v:v = 1:1) three times followed by drying *in vacuo* overnight to give 230.8 mg of orange yellow powder, poly(2-((2-(methacryloyloxy)ethyl)disulfanyl)ethyl ferrocene-carboxylate) (PFcMAss).

### 2.4. Preparation of PFcMAss-grafted Graphene Oxide Sheets (GO-PFcMAss)

GO-based initiator containing ATRP initiation groups (–COC(CH_3_)_2_Br), TRIS-GO-Ini, was obtained by treating tris(hydroxymethyl) aminomethane (TRIS)-modified GO with 2-bromo-2-methylpropionyl bromide according to previous reports [47,48]. CuBr (3.0 mg, 0.02 mmol), TRIS-GO-Ini (65.8 mg) and FcMAss (625.2 mg, 1 mmol) were added to a 25 mL Schlenk flask (flame-dried under vacuum prior to use) sealed with a rubber septum under N_2_. After three cycles of evacuating and purging with N_2_, 5 mL of dried toluene was charged via a gastight syringe and the mixture was sonicated for 30 min in a thermostatic water bath at 25 °C. After two cycles of freezing-pumping-thawing, PMDETA (4.3 μL, 0.02 mmol) and EBiB (3.3 μL, 0.02 mmol) were added and the flask was degassed by three cycles of freezing-pumping-thawing, followed by immersing the flask into an oil bath set at 50 °C. The polymerization lasted 2 days and it was terminated by putting the flask into liquid N_2_. After diluting with toluene, the reaction mixture was filtered through a 0.22 μm filter and washed exhaustively with toluene and deionized water until the filtrate turned clear.

The obtained black solid was dried overnight at 45 °C in vacuo so as to afford 55.2 mg of GO-PFcMAss. Meanwhile, the filtrate was passed through a short Al_2_O_3_ column to remove the residual copper complex and precipitated into cold *n*-hexane/diethyl ether (v/v = 1:1) three times. Subsequently, free PFcMAss homopolymer was obtained by drying *in vacuo* overnight at 45 °C. GPC: *M*_n_ = 13,600 g/mol, *M*_w_/*M*_n_ = 1.48. GO-PFcMAss: XPS (molecular molar ratio): C, 72.71%; N, 1.31%; O, 23.26%, Fe, 1.01%, S, 1.60%. FT-IR: *ν* (cm^−1^): 2958, 2852, 1708, 1639, 1593, 1459, 1400, 1271, 1138, 817.

### 2.5. Fabrication of PFcMAss-AuNPs

Nanoparticles were prepared by Brust-Schiffrin protocol with some modifications [49]. A solution containing tetraoctylammonium bromide (TOAB, J&K, 98%, 13.7 mg, 0.025 mmol) in 10 mL of toluene was mixed with a solution containing tetrachloroauric acid (HAuCl_4_·4H_2_O, Sinopharm, AR, 10.3 mg, 0.025 mmol) in 0.21 mL of deionized water. After stirring at room temperature for 1 h, two layers were observed: the upper organic phase was orange-yellow, while the lower aqueous phase was colorless. Next, a solution containing sodium borohydride (NaBH_4_, Alfa Aesar, 98%, 9.5 mg, 0.025 mmol) in 2 mL of deionized water was added dropwise, resulting in a color change of the upper organic phase to wine red. After the mixture was stirred for another 2 h, a solution containing PFcMAss homopolymer (16.4 mg, amount of –S–S– disulfide bond: 0.0375 mmol) in 2 mL of toluene was added to the separated organic phase followed by stirring overnight at room temperature. The organic phase was concentrated and then dispersed in a toluene/ethanol mixture (v/v = 2:3). The resultant was sonicated for 1 min and the precipitant was collected. The crude product was purified by centrifugation to give a wine-red solid of PFcMAss-AuNPs.

### 2.6. Fabrication of GO-PFcMAss-AuNPs Nanocomposite

Nanoparticles were prepared by Brust-Schiffrin protocol with some modifications [49]. A solution containing TOAB (13.7 mg, 0.025 mmol) in 10 mL of toluene was mixed with a solution containing HAuCl_4_·4H_2_O (10.3 mg, 0.025 mmol) in 0.21 mL of deionized water. After stirring at room temperature for 1 h, two layers were observed: the upper organic phase was orange-yellow, while the lower aqueous phase was colorless. Next, a solution containing NaBH_4_ (9.5 mg, 0.025 mmol) in 2 mL of deionized water was added dropwise, resulting in a color change of the upper organic phase to wine-red. After the mixture was stirred for another 2 h, the upper organic phase was separated. GO-PFcMAss (10 mg) was dispersed in 2 mL of toluene followed by sonication for 10 min. The dispersion was added to the separated organic phase followed by stirring overnight at room temperature. Afterward, the organic phase was diluted with toluene and then centrifiguated (6,000 rpm) to remove the unreacted AuNPs. The filtrate was concentrated and diluted with deionized water followed by centrifugation (6,000 rpm) to remove the residual TOAB. The residual mixture was lyophilized to give 31.5 mg of wine-red solid, i.e. GO-PFcMAss-AuNPs.

## 3. Results and Discussion

### 3.1. SynthesisofFcMAss Monomer and Preparation of FcMAss Homopolymer

Although thiol-based compounds have been widely used as anchoring groups via Au–S bond, the thiol group, which can serve as a chain transfer agent, will interfere with smooth process of living/controlled radical polymerization [50,51,52]. Therefore, thiol-containing monomer generally cannot be directly polymerized by these methods. Satisfyingly, Tong and co-workers reported that dialkyl disulfide also can serve as anchoring group to stabilized AuNPs, similar to thiol [53]. Therefore, a new methacrylate monomer of 2-((2-(methacryloyloxy)ethyl)disulfanyl)ethyl ferrocene- carboxylate (FcMAss) containing disulfide and Fc groups was designed and synthesized via the estification reaction between methacryloyl chloride and the intermediate which was obtained by the reaction of bis(2-hydroxyethyl) disulfide with ferrocenoyl chloride (Scheme 2). For this new monomer, disulfide serves as an anchoring group to stabilize AuNPs and Fc is used as a model moiety to demonstrate that disulfide group can serve as a linker to introduce some desired functional groups.

**Figure 1:**
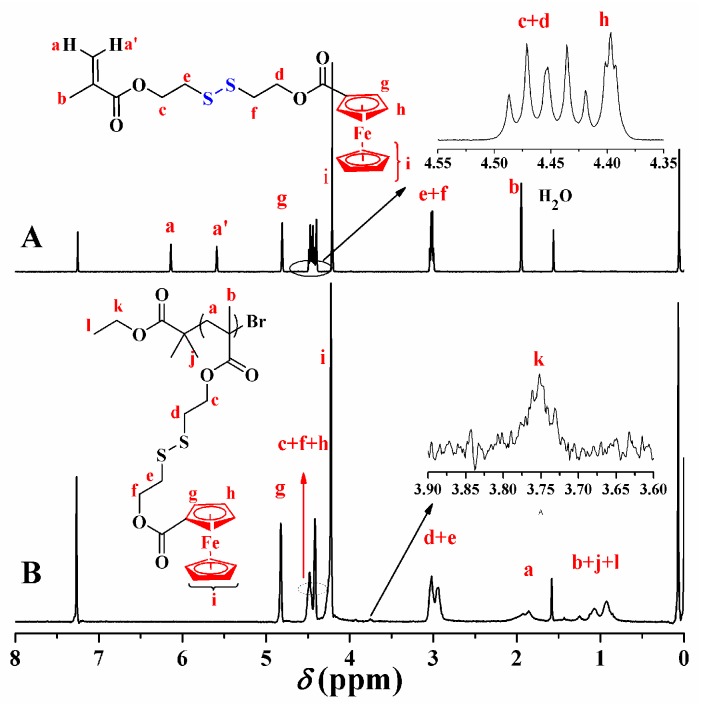
^1^H-NMR spectra of (**A**) FcMAss monomer and (**B**) PFcMAss homopolymer.

The chemical structure of FcMAss monomer was examined by FT-IR, ^1^H-NMR and MS. In Figure 1A, the proton resonance signals of double bond located at 6.14 (peak ‘a’) and 5.59 (peak ‘a’) ppm and 9 protons of Fc unit located at 4.81 (peak ‘g’), 4.40 (peak ‘h’) and 4.21 (peak ‘i’) ppm were found, and the ESI-MS result (434) was also consistent with the theoretical value. All these results confirmed the successful synthesis of FcMAss monomer.

Surface-modification for various materials can not only transform those materials with have excellent bulk physical and chemical properties into valuable materials with suitable surface properties required for specific applications, but also combine different functionalities of various materials to provide desired materials with multi-functionality or improved functionality. To extend the application of carbon nanomaterials, great efforts on the surface modification of carbon nanomaterials including fullerenes, carbon nanotubes, graphene and GO, have been made to improve the properties or introduce new functionality [54,55,56,57,58]. Among these methods, surface modification through grafting polymers is one of the most efficient ways to adjust surface functionality and properties. In the present work, SI-ATRP, which is considered to be one of the most versatile techniques to introduce functional polymeric chains onto the surface of matrices to prepare hybrid organic/inorganic materials [59,60,61,62], was used for preparation of PFcMAss-grafted graphene sheets. Before the surface functionalization of GO with PFcMAss by SI-ATRP, we firstly ran bulk ATRP of FcMAss by using ethyl α-bromoisobutyrate (EBiB) as initiator and CuBr/PMDETA as catalytic system at 50 °C to explore suitable polymerization conditions for SI-ATRP (Scheme 3). After polymerization for 2 days, GPC curve of the resulting PFcMAss homopolymer (Figure S1) shows only a unimodal and symmetrical eluent peak with a relatively narrow molecular weight distribution (*M*_w_/*M*_n_ = 1.33). Moreover, typical proton resonance signals of double bond also disappeared while characteristic peaks attributed to the protons of polymethacrylate backbone appeared at 0.75–1.26 and 1.87 ppm (peak ‘a’ and ‘b’) in ^1^H NMR spectrum after ATRP (Figure 1B). The preserving of signals attributed to Fc (peak ‘g’, ‘h’ and ‘i’) and methylene (peak ‘c’, ‘d’, ‘e’ and ‘i’) groups of FcMAss repeat unit illustrated that the structure of monomer remained intact during the polymerization. In addition, the appearance of a minor peak at 3.75 ppm (peak ‘l’) assigned to two protons of methylene moiety in the initiating group verified the mechanism of ATRP. The molecular weight of obtained PFcMAss homopolymer was estimated to be about 8,400 g/mol on the basis of integrations of peak ‘l’ and ‘g’ (*M*_n.NMR_ = *M*_ini_ + M*(*S*_g_/*S*_l_), *M*_ini_ and *M* are molecular weights of EBiB initiator and FcMAss monomer, respectively; *S*_l_ and *S*_g_ are integrations of peak ‘l’ and ‘g’, respectively), which is very closed to the value obtained by GPC (*M*_n_,_GPC_ = 8,600 g/mol). Thus, it is clear that FcMAss monomer could be successfully polymerized by ATRP using CuBr/PMDETA as catalytic system and EBiB as initiator at 50 °C.

### 3.2. Preparation of PFcMAss-Grafted Graphene Sheets (GO-PFcMAss)

According to our previous reports [47,48], ATRP initiating groups were introduced onto the surface of GO by treating GO with tris(hydroxymethyl) aminomethane (TRIS), followed by reacting with BrCOC(CH_3_)_2_Br. SI-ATRP of FcMAss was then conducted at 50 °C in toluene using Br-decorated GO as initiator and CuBr/PMDETA as catalytic system. EBiB was also added into the polymerization system as a sacrificial initiator for subsequent determination of molecular weight of PFcMAss chains grafted from GO, assuming that free polymers formed in the solution have the similar molecular weight as those formed on the surface [62,63,64].

**Figure 2:**
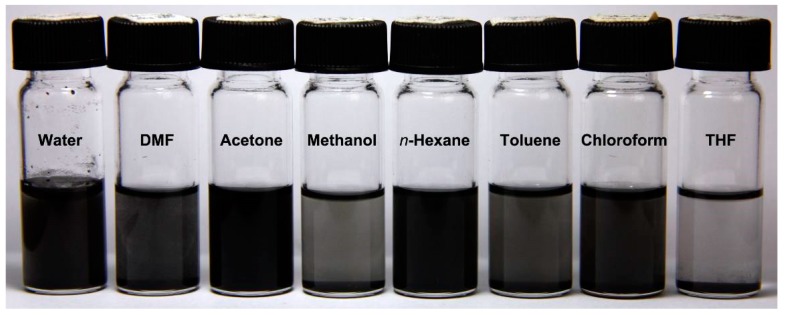
Digital photo of GO-PFcMAss in different organic solvents and water.

**Figure 3:**
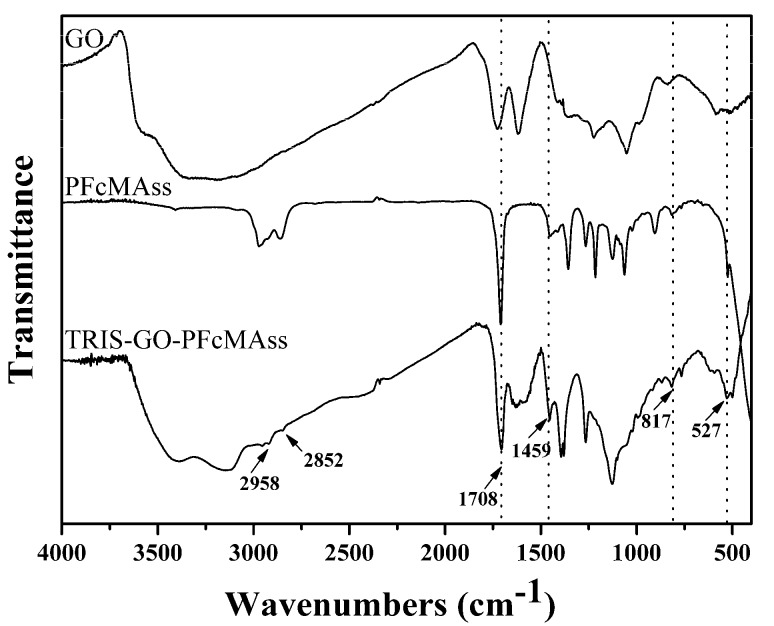
FT-IR spectra of GO, PFcMAss and GO-PFcMAss.

**Figure 4:**
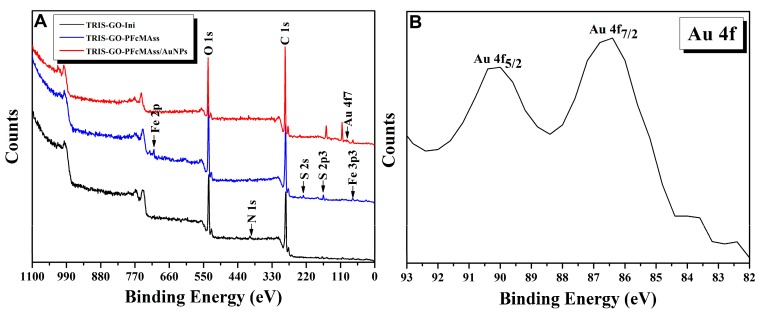
(**A**) XPS spectra of TRIS-GO-Ini, GO-PFcMAss and GO-PFcMAss/AuNPs. (**B**) XPS spectrum of Au4f in GO-PFcMAss-AuNPs.

The as-prepared GO-PFcMAss samples showed good dispersible properties in solvents such as acetone, chloroform, THF, DMF and toluene (Figure 2). However, they showed poor dispersibility in water and methanol, which are good solvents for GO, but poor solvents for PFcMAss (Figure 2A). From FT-IR spectra of GO before and after SI-ATRP (Figure 3), one can notice that typical signals of Fc group at 2958, 1459 and 817 cm^−1^ and a new peak at 2852 cm^−1^ attributed to stretching vibration of methylene group appeared, along with an obvious increase of the intensity of characteristic peak of carbonyl at 1708 cm^−1^. In addition, a characteristic disulphide mode at 527 cm^−1^ was observed, indicating the presence of disulfide bonds in the obtained GO-PFcMAss. From XPS spectra of pristine GO and functionalized GO after SI-ATRP (Figure 4A), two peaks at 709 and 71 eV were attributed to Fe 2p and Fe 3p3, along with two peaks at 229 and 165 eV originating from S 2s and S 2p3. From the elemental composition of obtained GO-PFcMAss obtained from XPS (Table S1), one can see that the content of Fe (mol%) is about 1.1 mol%, which meant that the weight content of PFcMAss in GO-PFcMAss composite is about 44.2 wt%. Moreover, GPC analysis showed that free PFcMAss homopolymer had a *M*_n_ of 13,600 g/mol with a *M*_w_/*M*_n_ of 1.48 (Figure S1). All of these observations indicated that PFcMAss chains with a *M*_n_ of 13,600 g/mol were introduced onto the surface of GO by SI-ATRP.

### 3.3. Fabrication of GO-PFcMAss-AuNPs Nanocomposite

Brust-Schiffrin method has been considered to be one of the most attractive approaches for preparing thiolate-protected gold nanoparticles using thiol-based ligands with extraordinary stability and potentials for further surface functionalization [49]. We then tested whether we could use PFcMAss with disulfide groups, instead of thiol-containing species, in the preparation of AuNPs via Brust-Schiffrin protocol [49]. In the present work, we also employed PFcMAss as stabilizer to prepare AuNPs by Brust-Schiffrin protocol with some modifications as outlined in Scheme S1. After Au(III) of AuCl_4_^−^ ions were reduced to Au(0) by NaBH_4_, the organic phase was firstly separated before the addition of PFcMAss so as to avoid the decomposition of PFcMAss caused by NaBH_4_ in aqueous phase. It is supposed that the surface of AuNPs would be coated by PFcMAss via ligand exchange since Au-S bond was stronger than the interaction between Au and N of tetraoctylammonium bromide (TOAB).

The as-prepared PFcMAss-AuNPs can be dispersed in chloroform, toluene, THF and ethanol to give transparent colloidal solution with a wine-red color. These solutions still remained stable over several months. On the contrary, purple aggregates of gold particles were formed in a control experiment, where PFcMAss homopolymer was not added (Figure S2). From ^1^H-NMR spectrum of PFcMAss-AuNPs powder obtained by centrifugation (Figure S3), one can notice the typical signals at 4.22, 4.41 and 4.82 ppm attributed to Fc group and peaks at about 2.85 and 3.12 ppm originating from the protons in the methylene spacer group. Additionally, the characteristic signals originating from Fc units (2958, 1459 and 817 cm^−1^), carbonyl (1708 cm^−1^) and methylene group (2923 and 2852 cm^−1^) of FcMAss repeat unit were found in FT-IR spectrum (Figure S4) of the obtained PFcMAss-AuNPs powder. All these evidences demonstrated that PFcMAss chains were attached on the surface of AuNPs.

**Figure 5:**
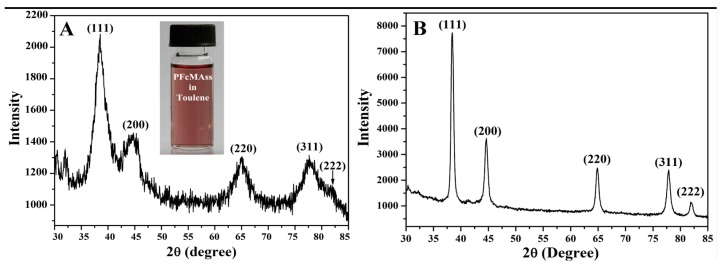
XRD pattern of PFcMAss-functionalized AuNPs (**A**) and GO-PFcMAss-AuNPs (**B**).

From XRD pattern of PFcMAss-AuNPs power obtained by centrifugation (Figure 5A), one can notice five diffraction peaks at scattering angles of 38.4°, 44.7°, 65.1°, 77.8° and 81.7°, corresponding to (111), (200), (220), (311) and (222) crystal planes of the face center cubic structure of AuNPs [46]. Based on XRD results, the average diameter of AuNPs was estimated to be about 4.5 nm according to Scherrer formula (*L*_hkl_ = kλ/βcosθ, where *L*_hkl_, λ, θ, β and k are the size of microcrystals perpendicular to face (hkl), wavenumber of X-ray, Bragg angle, diffraction line width and Scherrer factor, respectively).

**Figure 6:**
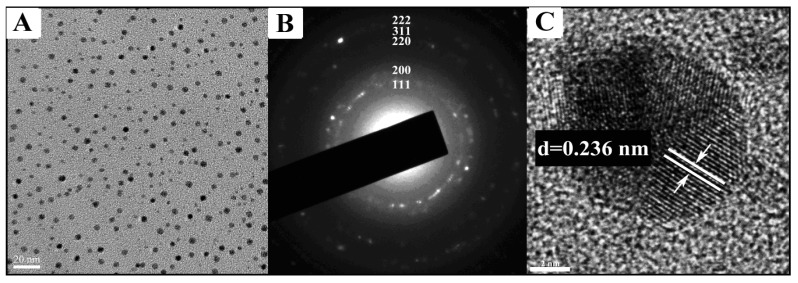
(**A**) TEM image, (**B**) selected area electron diffraction pattern and (**C**) HRTEM image and of PFcMAss-AuNPs.

As shown in TEM image of PFcMAss-AuNPs (Figure 6A), the obtained AuNPs are spherical and uniform in size with an average diameter of 4.7 nm, which is consistent with the result calculated from the Scherrer formula. A selected area electron diffraction (SAED) pattern of obtained AuNPs with a core size of 5.1 nm exhibits five concentric diffraction rings from inside to outside corresponding to (111), (200), (220), (311) and (222) crystal planes of AuNPs (Figure 3B), consistent with XRD results. In addition, the spacing value was found to be 0.238 nm from HRTEM image (Figure 3C), in good agreement with interplanars pacing of Au (111) [65]. Since TEM measurement generally only shows the morphology of Au nanoparticles, and the organic or polymeric ligands on the surface of gold nanoparticles are not able to be visualized due to their much lower electron density than that of gold nanoparticles [66]. However, both gold nanoparticle and ligand can be detected by AFM since AFM measurement mainly reflects the height of sample, regardless of electron density of the object. It can be found that the average diameter of spherical particles in AFM image was about 16 nm (Figure S5), much larger than the size obtained by TEM (4.7 nm). These results further made sure that AuNPs were coated with PFcMAss polymers. Thus, it can be concluded that PFcMAss homopolymer could serve as excellent stabilizer for the preparation of AuNPs with high quality by the optimized Brust-Schiffrin method.

On the basis of these results, we then attempted to functionalize GO-PFcMAss with AuNPs through depositing AuNPs onto the surface of GO-PFcMAss *in situ* via the modified Brust-Schiffrin strategy using GO-PFcMAss as stabilizer instead of PFcMAss homopolymer. Figure 5B displays XRD pattern of GO-PFcMAss decorated with AuNPs (GO-PFcMAss-AuNPs nanocomposites). Five obvious diffraction peaks appeared at scattering angles of 38.4°, 44.7°, 65.1°, 77.8° and 81.7°, corresponding to (111), (200), (220), (311) and (222) crystal planes, respectively, which is indicative of the presence of AuNPs in the as-prepared nanocomposites. The average diameter of formed AuNPs was calculated to be about 3.8 nm on the basis of XRD results and Scherrer formula. In the XPS spectrum of GO-PFcMAss-AuNPs nanocomposite (Figure 4A), the Fe3p3 peak at a binding energy value of 71 eV was observed, suggesting the presence of ferrocene unit on GO sheets. It can be seen that the C1s peak attributed to GO appeared at a binding energy value of 285.7 eV. The appearing of Au 4f doublet (86 and 90 eV, in Figure 4B) was consistent with the Au^0^ state, revealing the presence of AuNPs on graphene sheets. Furthermore, the element contents of TRIS-GO-Ini, GO-PFcMAss and GO-PFcMAss-AuNPs based on XPS results were obtained as outline in Table S1, indicating the existence of C, N, O, Fe, S and Au elements in GO-PFcMAss-AuNPs. According to previous literatures [67,68], there are two sulfur species in organic layer absorbed on the surface of AuNPs. The weight content of Au in the obtained GO-PFcMAss-AuNPs nanocomposite was estimated to be 1.1 wt% based on the elemental composition of GO-PFcMAss-AuNPs (Table S1). In addition, the contents of Fe and S decreased from 1.01 mol% and 1.60 mol% to 0.04 mol% and 0.38 mol%, respectively, after the introduction of AuNPs. The decrease of contents of Fe and S, especially the decrease of ratio of content of Fe to content of S, indicated that part of disulfide groups might be broken during the formation of AuNPs to give FcCH_2_CH_2_S-moieties and part of produced FcCH_2_CH_2_S-species were lost during the preparation of GO-PFcMAss-AuNPs nanocomposite. However, there was about 1.0 wt% of Fc units in GO-PFcMAss-AuNPs nanocomposite.

**Figure 7:**
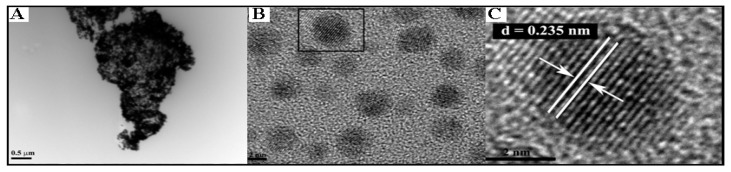
(**A**) TEM, (**B**) and (**C**) HRTEM images of GO-PFcMAss-AuNPs nanocomposite.

From TEM image (Figure 7A), one can notice that there were a large number of AuNPs located on the surface of GO and almost no free AuNPs can be found. These observations indicated that PFcMAss of GO-PFcMAss had a high affinity to capture any free AuNPs formed in the solution via strong S-Au interactions and PFcMAss domains on the surface of GO served as excellent nucleation sites for the growth of AuNPs *in situ*. The size of formed AuNPs was about 4.1 nm, which was close to the value estimated from XRD result (3.8 nm). The spacing value of one gold nanoparticle was estimated to be about 0.235 nm (Figure 7C), in good agreement with interplanar spacing of Au (111) [65], which further verified the formation of AuNPs on the surface of GO-PFcMAss. Therefore, all these results indicated that GO-PFcMAss-AuNPs nanocomposite can be prepared by using GO-PFcMAss as stabilizer, instead of organic ligand, via modified Brust-Schiffrin method.

## 4. Conclusions

In summary, we developed a facile and versatile strategy to prepare multi-functional GO-based nanocomposites containing AuNPs and Fc groups. A new methacrylate monomer of FcMAss containing both disulfide and Fc groups was designed and synthesized. This monomer can be polymerized by ATRP using CuBr/PMDETA as catalytic system and EBiB as initiator at 50 °C. PFcMAss polymer chains were then efficiently introduced onto the surface of GO by SI-ATRP using Br-containing GO as macroinitiator. By taking advantage of strong coordinating ability of disulfide moieties toward AuNPs, a large number of AuNPs with an average diameter of about 4.1 nm were formed *in situ* on the surface of PFcMAss-decorated GO (GO-PFcMAss) via modified Brust- Schiffrin strategy to give multifunctional GO-PFcMAss-AuNPs nanocomposite consisting of GO, AuNPs and Fc groups. The usage of disulfide-containing polymers, instead of commonly used thiol-based compounds, not only endowed GO-PFcMAss-AuNPs nanocomposites with excellent dispersibility and colloidal stability, but also facilitated the introduction of much more AuNPS onto the surface of GO due to a high local density of disulfide groups. The polymeric chains on the surface of GO endowed GO-PFcMAss-AuNPs nanocomposite with excellent colloidal stability. Additionally, the disulfide group of PFcMAss provided potential sites to efficiently incorporate additional functionalities by easily modifying structure of disulfide-based monomer. Significantly, given attractive properties and broad applications of GO-, AuNPs- and Fc-based materials, this kind of nanocomposite undoubtedly shows promising applications in the fabrication of multi-functional materials.

## Supplementary Materials

The following are available online at https://www.mdpi.com/2079-4991/9/2/310/s1, Figure S1: GPC curves of PFcMAss homopolymer and free PFcMAss polymer during surface-initiated ATRP, Table S1: Elemental analysis of TRIS-GO-Ini, GO-PFcMAss, and GO-PFcMAss-AuNPs obtained from XPS, Figure S2: Photos of solutions of AuNPs prepared by Brust-Schiffrin protocol with (A) and without (B) the addition of TRIS-GO-PFcMAss, Figure S3: ^1^H NMR spectra of PFcMAss and PFcMAss-AuNPs in CDCl_3_ and the photo (inset) of PFcMAss in toluene (0.1 mg/mL), Figure S4: FT-IR spectra of PFcMAss and PFcMAss-AuNPs, Figure S5: Tapping mode AFM images with section analysis of PFcMAss-functionalized AuNPs.
