# Supplementary material for: Graphene Oxide/Ferrocene-Containing Polymer/Gold Nanoparticle Triple Nanocomposite
Source: Nanomaterials (Basel). 2019 Feb 25;9(2):310. doi: 10.3390/nano9020310 (PMC6410150; doi:10.3390/nano9020310)
Supplement: Supplementary file 1 [file nanomaterials-09-00310-s001.pdf]

Supporting Information

Graphene Oxide/Ferrocene-containing Polymer/Gold Nanoparticle Triple Nanocomposite

Wenhao Qian [1,*], Tao Song [1], Mao Ye [1], Haiyan Zhang [1], Chun Feng [2], Guolin Lu [2] and Xiaoyu Huang [2,*]

[1] Department of Stomatology, Shanghai Xuhui District Dental Center, 685 Zhaojiabang Road, Shanghai 200032, China; songtao21@139.com (T.S.); doreye@139.com (M.Y.); 13641956377@163.com (H.Z.)

[2] Key Laboratory of Synthetic and Self-Assembly Chemistry for Organic Functional Molecules, Center for Excellence in Molecular Synthesis, Shanghai Institute of Organic Chemistry, Chinese Academy of Sciences, 345 Lingling Road, Shanghai 200032, China; cfeng@sioc.ac.cn (C.F.); luguolin@sioc.ac.cn (G.L.)

* Correspondence: pingyanlaoto@163.com (W.Q.); xyhuang@sioc.ac.cn (X.H.); Tel.: +86-21-64045643 (W.Q.); +86-21-54925310 (X.H.)

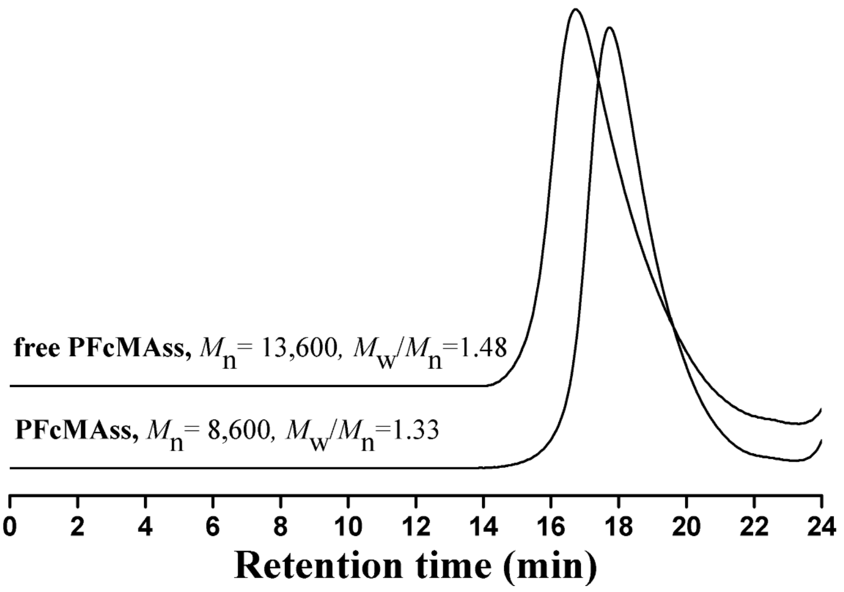

Figure 1. GPC curves of PFcMAss homopolymer and free PFcMAss polymer during surface-initiated ATRP.

Table 1. Elemental analysis of TRIS-GO-Ini, GO-PFcMAss and GO-PFcMAss- AuNPs[a].

Sample	C% (mol%)	N% (mol%)	O% (mol%)	Fe% (mol%)	S% (mol%)	Au% (mol%)
TRIS-GO-Ini	79.51	1.83	17.48	NA	NA	NA
GO-PFcMAss	72.71	1.31	23.26	1.01	1.60	NA
GO-PFcMAss-AuNPs	67.67	0.63	17.03	0.04	0.38	0.06

[a] Obtained from XPS.

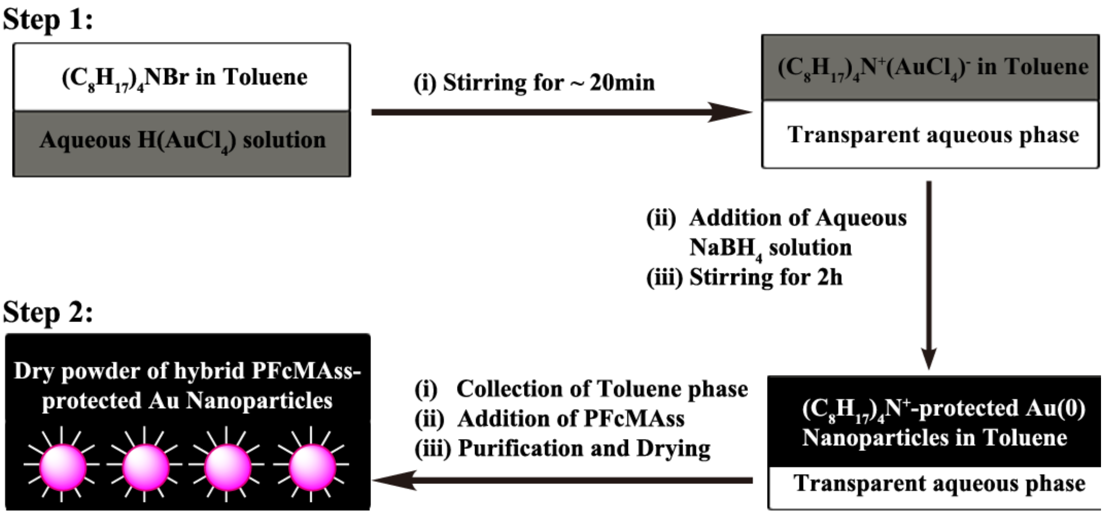

Scheme S1. Preparation of PFcMAss-protected AuNPs by optimized Brust-Schiffrin approach.

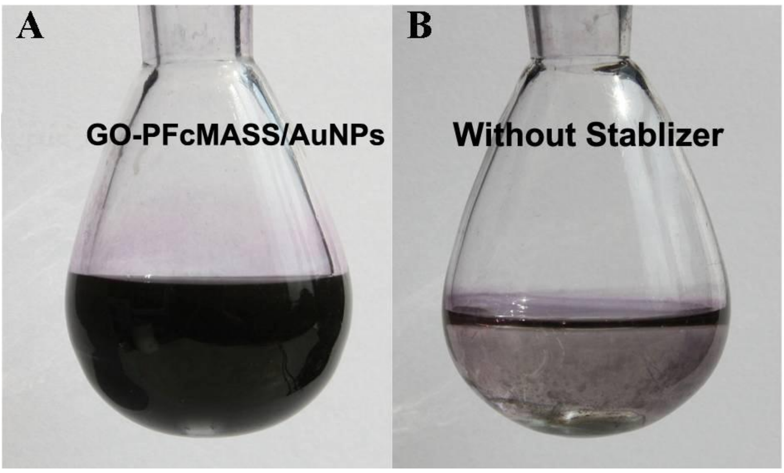

Figure 2. Photos of solutions of AuNPs prepared by Brust-Schiffrin protocol with (A) and without (B) the addition of GO-PFcMAss.

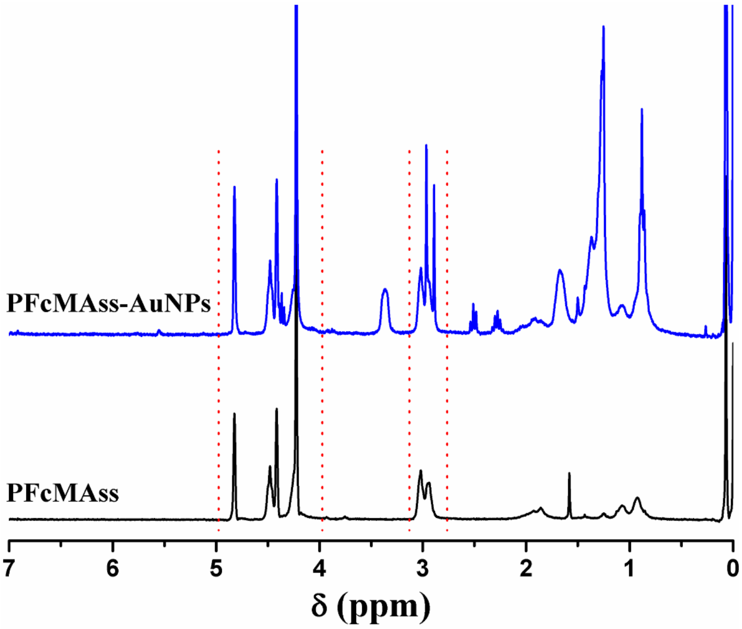

Figure 3. ^{1}H-NMR spectra of PFcMAss and PFcMAss-AuNPs in $CDCl_3$.

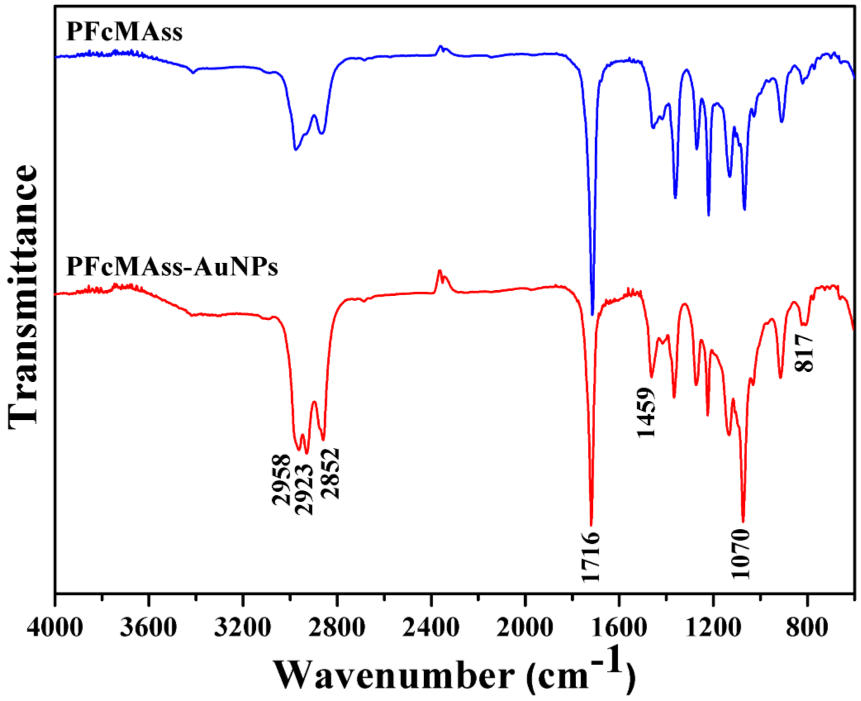

Figure 4. FT-IR spectra of PFcMAss and PFcMAss-AuNPs.

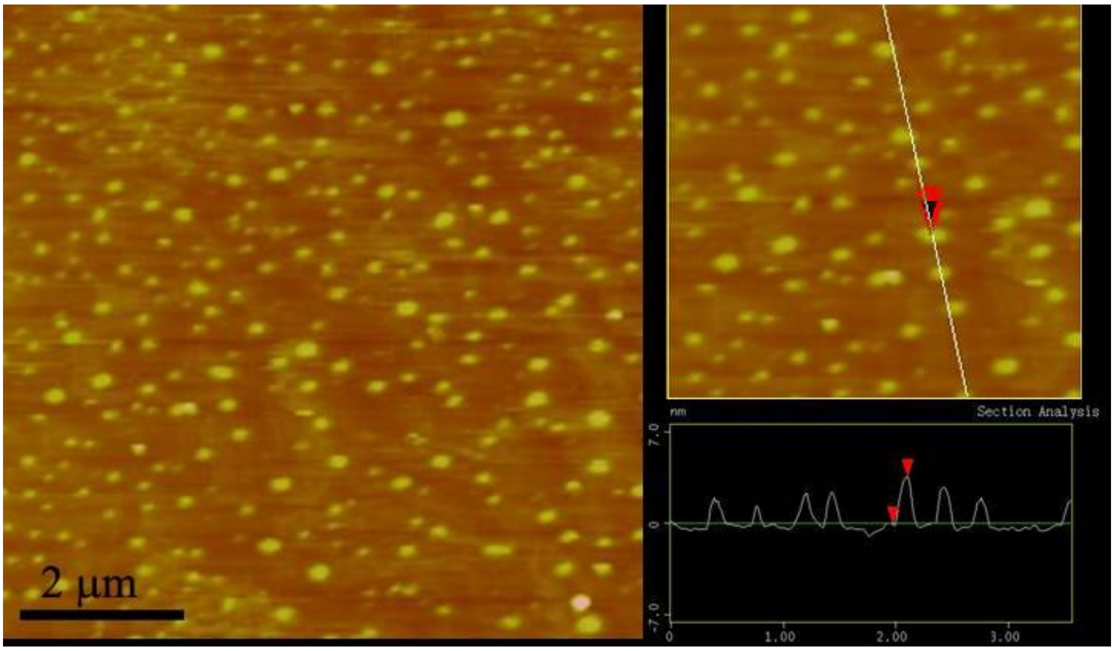

Figure 5. Tapping mode AFM images with section analysis of PFcMAss- functionalized AuNPs.
